# Eco-Friendly Sustainable Concrete and Mortar Using Coal Dust Waste

**DOI:** 10.3390/ma16196604

**Published:** 2023-10-09

**Authors:** Evgenii M. Shcherban’, Sergey A. Stel’makh, Alexey N. Beskopylny, Levon R. Mailyan, Besarion Meskhi, Diana Elshaeva, Andrei Chernil’nik, Alexander L. Mailyan, Oxana Ananova

**Affiliations:** 1 Department of Engineering Geology, Bases and Foundations, Don State Technical University, 344003 Rostov-on-Don, Russia; au-geen@mail.ru; 2 Department of Unique Buildings and Constructions Engineering, Don State Technical University, 344003 Rostov-on-Don, Russia; sergej.stelmax@mail.ru (S.A.S.); lrm@aaanet.ru (L.R.M.); diana.elshaeva@yandex.ru (D.E.); chernila_a@mail.ru (A.C.); 3 Department of Transport Systems, Faculty of Roads and Transport Systems, Don State Technical University, 344003 Rostov-on-Don, Russia; 4 Department of Life Safety and Environmental Protection, Faculty of Life Safety and Environmental Engineering, Don State Technical University, 344003 Rostov-on-Don, Russia; spu-02@donstu.ru; 5 Department of Urban Construction and Economy, Don State Technical University, 344003 Rostov-on-Don, Russia; mailyan_a@sroufo.ru; 6 Department of Marketing and Engineering Economics, Faculty of Innovative Business and Management, Don State Technical University, 344003 Rostov-on-Don, Russia; o_ananova@mail.ru

**Keywords:** coal dust, concrete, mortar, compressive strength, bending strength, water absorption

## Abstract

Finding the solution to the problem of the accumulating waste from the mining and processing industries, as well as reducing their carbon footprint, is among the most important tasks today. Within the construction industry, in the field of the production of building materials such as concrete, these problems may be solved through the use of waste and by saving the binder component. The purpose of this study is to substantiate the feasibility of using waste coal dust (CD) in concrete and cement–sand mortars as a partial replacement for cement. Test samples were made by partially replacing cement with CD in an amount from 0% to 10% in increments of 2% by weight. The following main characteristics were studied: mobility and density of mixtures, as well as density, compressive strength, bending strength and water absorption of concrete and mortars. X-ray diffraction and microscopic analysis methods were used in this work. The introduction of CD to replace part of the cement, up to 10%, did not have a significant effect on the density of concrete and mortar mixtures but reduced their workability. The best values of physical and mechanical characteristics were recorded for concrete and mortar with 4% CD. The increases in the compressive strength of concrete and mortars were 6.6% and 5.7%, and in flexural strength 6.1% and 5.6%, respectively. Water absorption decreased by 9.7% for concrete and by 9.3% for mortar.

## 1. Introduction

Currently, in many countries of the world, there is a big problem associated with the accumulation of waste from the mining and processing industries [1,2,3,4,5], in particular, for industrial mining purposes. For example, in coal mines and related industries, a huge amount of by-material of coal products is formed that is not used for its intended purpose [6,7]. One of the most typical representatives of this waste group is coal dust. This problem is inherent in many countries and regions of the world where the mining industry is well developed. Enterprises and scientific institutes in such countries face a serious challenge in developing proposals at the fundamental and applied levels to study the possibility of using such coal dust [8,9,10].

Coal dust can be classified as an inert mineral additive that does not fully react with the binder component in the concrete mixture. The main purpose of using additives of this type in concrete technology is the additional compaction of cement composites achieved by reducing porosity [11,12]. These types of additives include clay, metakaolin, electric arc furnace dust waste, various types of rock dust and finely ground calcium powders from aquaculture waste. For example, in the study [13], compositions of self-compacting concrete mixtures were developed in which part of the cement was replaced by calcined clay. It was found that when replacing up to 20% by volume, the fluidity of the mixture, compressive strength and durability of the developed composites did not change much and were comparable to the control composition. There is known work using the addition of metakaolin in self-compacting concrete (SCC) compositions [14]. As a result, it was found that with the optimal replacement of 10% of cement with metakaolin, SCC has good resistance to magnesium sulfate solution, chloride diffusion and water absorption, and the microstructure of these composites is characterized by a smaller amount of ettringite. A similar study was carried out in [15]. It was established that metakaolin in SCC at a rational quantity also has a positive effect on the strength, water absorption and porosity. The use of metakaolin as a partial replacement for cement in concrete technology was also studied in detail in [16,17,18,19]. It is noted that the use of this additive in rational quantities makes it possible to improve the strength characteristics and durability of composites and reduce their porosity and water absorption.

In studies [20,21,22,23,24,25,26,27,28], the use of various types of dust was investigated. The use of electric arc furnace dust in [20] when replacing part of the cement in an amount of 2% helped to improve the strength characteristics. In the study [21], the most effective concrete compositions were obtained by replacing part of the cement with freshly homogenized electric arc furnace dust in an amount of 4%. In [22], in addition to improving the mechanical properties of the composites, the results of SEM analysis showed that the introduction of electric arc furnace dust led to a noticeable decrease in ettringite crystals. The use of marble dust as a partial replacement for cement [23] in an amount of 10% increased the compressive, tensile and flexural strength by 12.68%, 21.71% and 16.73%, respectively, compared to the control mixture. Similarly, in [24], cement composites with 10% replacement of part of the binder with marble dust showed good results. In general, the effectiveness of using stone dust in concrete as a partial replacement for binders was also confirmed by the following works [25,26,27]. Powders from aquaculture waste are often used as a replacement for cement. For example, in [29], the use of finely ground oyster shell powder in an amount of 3% helped improve the properties of cement composites. And in work [30], when replacing part of the cement with seashell powder in an amount of 5–15%, the concrete had less porosity compared to control compositions. The positive effects of using finely ground powders of various types of shells as a replacement for part of the cement in concrete are also described in a number of the following studies [31,32,33].

The use of coal dust in building materials and in particular in cement composites was considered in works [8,9,10,34]. Overall, the addition of coal dust in certain quantities can facilitate its efficient use in the production of cement and concrete and thus make a significant contribution to the sustainable development of the construction industry in terms of long-term energy savings, a reduction in greenhouse gas emissions and the mitigation of global climate change [8]. These few studies made it possible to conduct a more accurate analysis of the current existing gaps in the study of the use of coal dust in concrete.

The relevance of the research being carried out is confirmed by the high degree of risk of violating the environmental agenda due to the accumulation of coal dust. Such dust has a direct impact on the creation of a carbon footprint and the risk of not achieving sustainable development goals. As for the relevance of the scientific research of this work, it should be noted that the natural mechanism for the formation of concrete based on coal dust is complex [34]. Coal dust, as a fine powdery material, has a certain chemical composition that must be carefully taken into account when integrating such a powdered material into the composition of a concrete conglomerate. In particular, it is necessary to clearly understand the physicochemical processes that occur during the formation of the structure of concrete based on coal dust and to evaluate the compatibility of coal dust with other components in the concrete [9,35]. In this case, one needs to clearly understand which component or additional component this powder material is replacing. The shape of these particles plays a significant role, as does their size and distribution, according to fractional analysis. X-ray phase analysis, carried out before using such coal dust in concrete, is important. Eliminating such scientific deficiencies will help to better assess the fundamental relationship between the composition, structure and properties of new concretes [36]. One way or another, any composite that uses a new, uncharacteristic component will be a rather unpredictable or hard-to-predict material. It is necessary to take into account not only the chemical, physical–mechanical and other characteristics of the raw materials but also aspects of concrete mixing and aspects of the distribution of coal dust in the concrete body in order to achieve maximum uniformity of particle distribution in combination with other components [37,38]. It is important to have a clear understanding of the role of these particles. This is necessary to prevent the creation of harmful clusters in which particles stick to each other and to strive for a uniform distribution of coal dust particles mixed with other components. That is why it is important to understand what the rational amount of coal dust in the body of the concrete is and how the required percentage of coal dust relates to the amount of other powdered materials, for example, cement or other fillers used simultaneously with coal dust. The compatibility of coal dust with fillers and concrete aggregates is important, for which the shape, development of the grain surface and other aspects that can affect the achievement or nonachievement of specified characteristics also play an important role [9,39]. Restrictions on the use of coal dust are regulated by technological and regulatory requirements. In terms of technology, the limitations arise from the need to comply with technological processes and rely on the requirements of standards. In the regulatory and technical sphere, limitations are determined by the lack of a systematized base of regulatory and technical documents, which gives such composites the character of experimental ones, requiring more serious justification and testing [40].

The purpose of this study is to substantiate the feasibility of using waste coal dust (CD) in concrete and cement–sand mortars as a partial replacement for cement. It is important to prevent a decrease in the characteristics of concrete when using coal dust compared to control samples. This effect can definitely be called positive and, based on this, our scientific hypothesis is formulated as follows. Provided that optimal recipe–technological factors and parameters are observed when achieving the compatibility of the components of concrete containing coal dust and the conditions for the formation of a rational structure, it is possible to achieve high-performance indicators of concrete that are at least not inferior to analogous concrete made without the use of such dust. The environmental effect of this development is achieved by creating ways to dispose of waste such as coal dust. In the same context, there is also an economic effect that makes it possible to reduce the cost of concrete by saving an expensive component—cement. The technological effect should be to prevent a decrease in the technological properties of the created carbon–concrete mixture. The scientific novelty of the ongoing research is the acquisition of new fundamental and applied knowledge about the patterns of formation of the structure and properties of concrete based on coal dust, depending on rational recipe–technological factors and parameters, as well as the achievement of empirical values within certain limits and ranges for the specified parameters.

## 2. Materials and Methods

In order to structure this research and present it in the form of a program, we first list the most key objects and parameters to be studied in order to study the effect of coal dust on cement composites. Based on the fact that coal dust is a finely dispersed modifier for cement composites, we will focus on such composites as cement mortars and heavy concrete. The choice of these research objects was made because these composites cover the widest field of application in modern construction. And in particular, in those areas where the extraction of the corresponding raw materials in the form of coal dust occurs, it is advisable to study these types of composites. At the same time, it is important to understand that it is not only hardened composites in finished form that are subject to research but also their fresh mixtures, which have a number of rheological and other technological characteristics. Thus, the experimental research program will include the most important and indicative properties and characteristics of mortar mixtures, cement mortars and concrete mixtures, that is, fresh concrete and hardened concrete. The parameters that determine the most important aspects of the influence of the addition of coal dust on the properties of all these mixtures and composites will be those properties that are strictly regulated in the regulatory and technical documentation and are the most important. These are the mobility of fresh composites and the mechanical strength characteristics of hardened composites.

### 2.1. Materials

The following raw materials were used as components for the manufacture of the experimental samples:

- Portland cement CEM I 52.5N (C) (CEMROS, Stary Oskol, Russia) [41];
- Crushed sandstone (CS) (RostMed, Kamensk, Russia);
- Quartz sand (S) (RostStroyMix, Rostov-on-Don, Russia);
- Coal dust (CD) (IMPEX-GROUP, Krasny Sulin, Russia).

The characteristics of the raw materials are presented in Table 1.

The plasticizer Sikament^®^ BV 3M (P) (Zika, Lobnya, Russia) was used as an additive to regulate the workability of the concrete and mortar mixtures.

**Figure 1:**
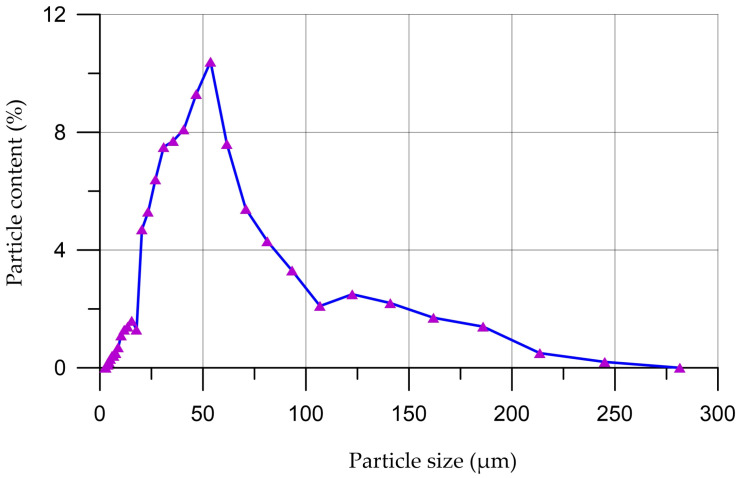
Distribution curve of coal dust particles.

**Figure 2:**
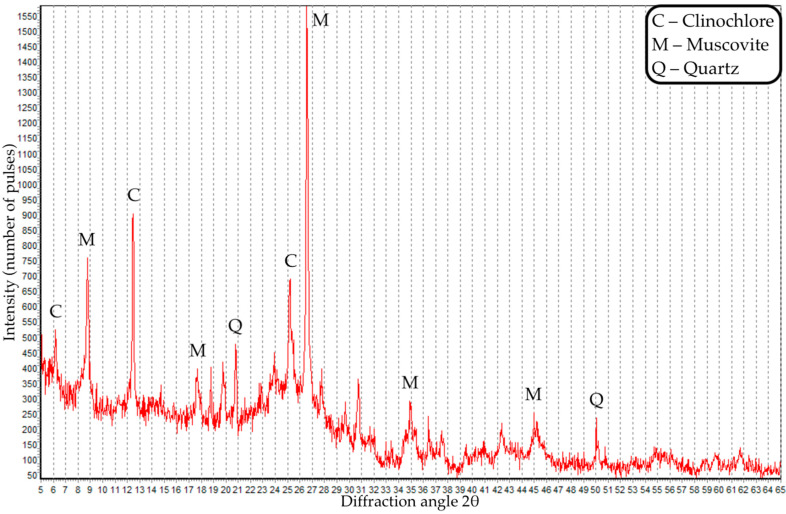
X-ray pattern of coal dust.

The grain composition and X-ray diffraction pattern of coal dust are presented in Figure 1 and Figure 2.

According to the granulometric curve presented in Figure 1, the largest proportion of coal dust particles, 67%, have sizes from 20 µm to 70 µm, the proportion of particles with a size of less than 20 µm is 9.4% and particles with a size of more than 70 µm is 23.6%.

Based on the results of XRD analysis of coal dust, we identified muscovite, quartz and chlorite phases, and an amorphous carbon phase was also present.

### 2.2. Methods

The compositions of experimental concrete mixtures and mortars are presented in Table 2 and Table 3.

The production of concrete and mortar samples included the following main technological steps. First, all raw materials were measured in accordance with the recipe. The production of concrete mixtures was carried out in a laboratory concrete mixer. All raw materials were premixed in dry form, and then mixing water with a plasticizing additive was introduced. Next, the concrete mixture was mixed until homogeneous and placed into molds, which were then additionally vibrated for 60 s.

A day later, all samples were removed from the molds and placed in a normal hardening chamber for 27 days at a temperature of 20 ± 2 °C and a relative humidity of 95%. The preparation of mortar samples was carried out in a similar way. After production, the mortar samples were stored in a normal hardening chamber for 27 days.

No differences were observed in the quality or macrostructure of the two types of concrete samples and mortar. The only striking difference was the color of the samples. Samples with coal dust had a pronounced black color.

The main technological equipment for the production of the concrete and mortar samples was as follows:

- Laboratory concrete mixer BL-10 (ZZBO, Zlatoust, Russia);
- Forms 2FK-100, 3FK-70, FB-400, 3FB40 (RNPO RusPribor, St. Petersburg, Russia);
- Normal curing chamber KNT-1 (RNPO RusPribor, St. Petersburg, Russia);
- Laboratory vibration platform (IMash, Armavir, Russia).

**Figure 3:**
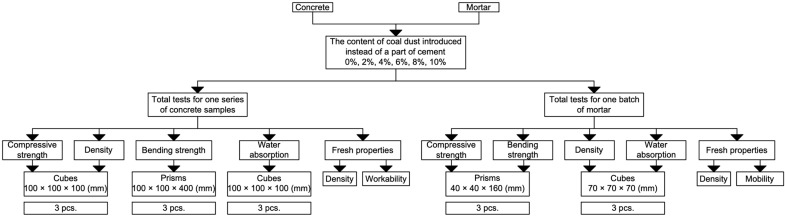
Experimental program.

The experimental program is presented in Figure 3.

A total of 36 cubes and 18 prisms of concrete as well as 18 cubes and 18 prisms of mortar were produced.

The fresh properties of the concrete mixture, such as density and workability, were determined in accordance with the methods of [42,43]. The concrete mixture was compacted in a rigid waterproof container with a known volume and mass and then weighed. Density was calculated using the formula

$$D=\frac{m_{1}-m_{2}}{V}$$

where *D* is the density of the concrete mixture (kg/m^3^); *m*_1_—mass of the container filled with compacted concrete (kg); *m*_2_—empty container mass (kg); *V*—capacity volume (m^3^).

The essence of the method for determining the settlement of a cone when testing a concrete mixture for mobility was to compact the prepared concrete mixture in the form of a truncated cone, and the measured distance to which the concrete mixture settled after removing the cone indicated the mobility of the concrete mixture.

Determination of the compressive strength of concrete was carried out in accordance with the methods in [44,45,46,47,48,49] using a Press P-50 installation (PKC ZIM, Armavir, Russia).

The density of hardened concrete was determined according to the method [50,51] as the ratio of the mass of concrete (sample) in a state of normal humidity, when the samples were stored for 28 days in a normal hardening chamber at a relative air humidity of at least 95% and a temperature of 18 to 22 °C, to its total volume.

The determination of water absorption of concrete samples was carried out in accordance with the requirements of regulatory documents [52,53]. The samples were placed in a container filled with water in such a way that the water level in the container was approximately 50 mm above the top level of the stacked samples. The water temperature in the container was (20 ± 2) °C. The samples were weighed every 24 h during water absorption with an error of no more than 0.1%. The mass of water flowing out of the pores of the sample onto the scale was included in the mass of the saturated sample. The test was carried out until the results of two consecutive weighings differed by no more than 0.1%. The samples were tested in a state of natural humidity. The water absorption of concrete of each sample (wt.%) was calculated with an error of up to 0.1% using the formula:

$$W=\frac{m_{w}-m_{d}}{m_{d}}\times 100$$

where *m_w_* is the mass of the sample saturated with water (g); *m_d_* is the mass of the dry sample (g).

The determination of the fresh properties of mortar mixtures, as well as the density of the hardened mortar, their compressive strength, bending strength and water absorption was carried out in accordance following the requirements of [54,55].

The mobility of the mortar mixture was characterized by the depth of immersion of the reference cone into it, measured in centimeters. The immersion depth of the cone was estimated based on the results of the arithmetic average of two tests on different samples of the mortar mixture from one batch.

The density of the mortar mixture was characterized by the ratio of the mass of the compacted mortar mixture to its volume and was expressed in kg/m^3^.

The strength of the hardened solution was determined using prism samples (bending strength) and then using half-prism samples obtained after destruction (compressive strength). The bending strength was calculated as the arithmetic mean of the two highest test results of three samples. The six prism halves obtained after the bending test were immediately subjected to a compression test. One half of the prism was placed between two plates in such a way that the side faces, which, during manufacturing, were adjacent to the walls of the mold, were on the planes of the plates, and the stops of the plates were tightly adjacent to the smooth end plane of the sample. The sample, together with the plates, was centered on the press base plate. The average rate of load increase during testing was (2.0 ± 0.5) MPa/s. The compressive strength of an individual sample was calculated as the quotient of the breaking load divided by the working area of the plate. The compressive strength was calculated as the arithmetic mean of the four highest test results of six samples.

The density of the solution was determined by testing cube samples with an edge of 70 mm. The samples were stored for 28 days in a normal hardening chamber at a relative air humidity of at least 95% and a temperature of (20 ± 2) °C.

The water absorption of the solution was determined using samples that were placed in a container filled with water in such a way that the water level was approximately 50 mm above the top level of the laid samples. Samples were weighed after every 24 h of water absorption. In general, the test parameters were the same as when determining the water absorption of concrete samples [54].

Particle size gradations of components of concrete and mortar were determined using a Microsizer model 201C (VA Insalt, St. Petersburg, Russia).

The chemical composition of fine coal dust was determined in accordance with the requirements of GOST R 55661-2013: Solid mineral fuel. Determination of ash content followed method NSAM 138-X: Chemical methods, Method of quantitative chemical analysis. Accelerated methods for determining rock-forming elements in rocks and ores followed NSAM 3-X methodology. Determination of the total sulfur content in rocks, ores and products of their processing was completed using the gravimetric method. For this purpose, the following equipment was used: analytical balance CAS CAUX-220 (Seoul, Republic of Korea), muffle furnace LOIP (St. Petersburg, Russia), low-temperature furnace SNOL (AB “UMEGA GROUP”, Utena, Lithuania) and spectrophotometer UNICO 2100 (Dayton, NJ, USA).

X-ray diffraction analysis was performed on an ARLX’TRA diffractometer using the characteristic radiation of a copper anode (wavelength CuKα11.5406 Å, CuKα21.5444 Å). The PDF-2 X-ray database was used to identify serpentinite phases and minor minerals.

Cured concrete and mortar samples retained a significant difference in color between the control composition and the CD composition. The appearance of the samples did not differ significantly, but in the control mortar samples, there were quite large surface pores.

## 3. Results

**Figure 4:**
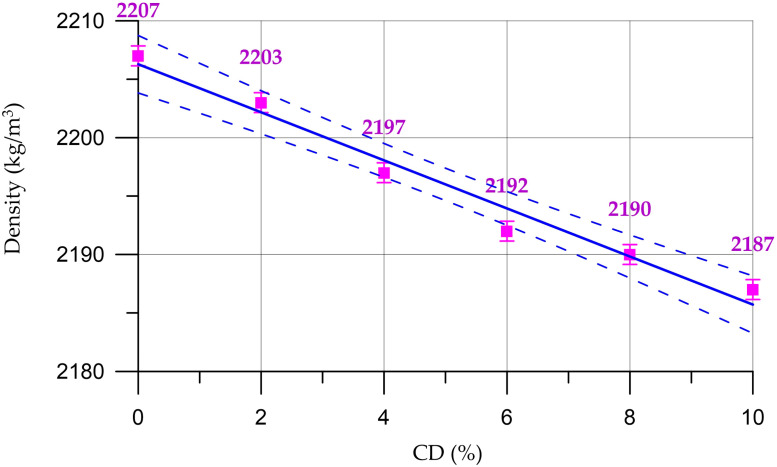
Dependence of the density of the concrete mixture on the CD content (the dashed line shows confidence limits with a level of 0.95).

**Figure 5:**
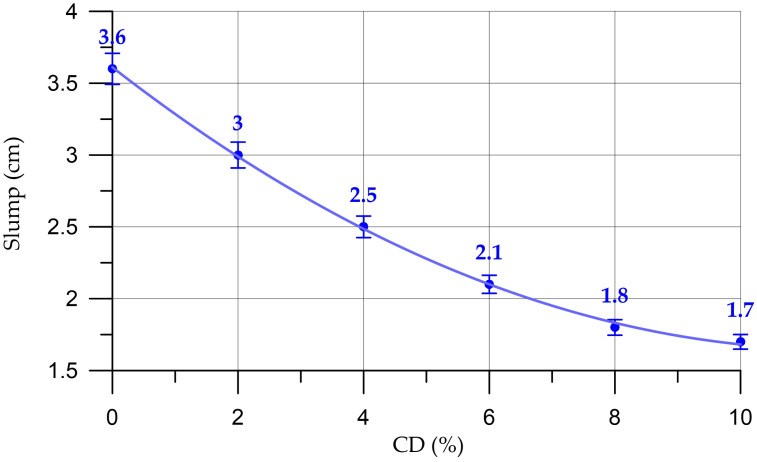
Dependence of mobility of concrete mixture on CD content.

**Figure 6:**
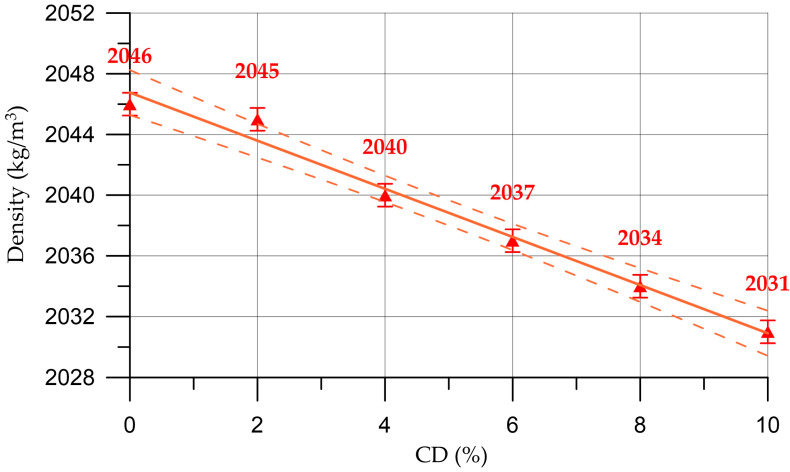
Change in mortar density depending on CD content (the dashed line shows confidence limits with a level of 0.95).

**Figure 7:**
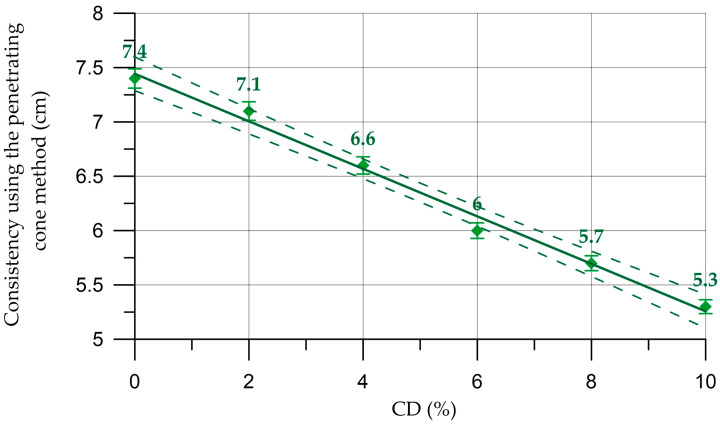
Change in mortar mobility depending on CD content (the dashed line shows confidence limits with a level of 0.95).

The results of this study of fresh properties of concrete and mortar mixtures are presented in Figure 4, Figure 5, Figure 6 and Figure 7.

The dependence of the density of the concrete mixture on the CD content (*x* in equation) presented in Figure 4 is described by a linear equation with a coefficient of determination *R*^2^:

(1) $$D_{cm}=2206.2-2.057\;x,\;\;\;\;R^{2}=0.975$$

The dependence of the mobility of the concrete mixture on the CD content (%) presented in Figure 5 is described by a second-degree equation:

(2) $$M_{cm}=3.61-0.34\;x+0.0147\;x,\;\;\;\;\;R^{2}=0.99$$

According to the results of the fresh properties of concrete mixtures, it was found that the density of concrete mixtures with an increase in the percentage of replacing part of the cement with coal dust decreases slightly and varies from 2207 kg/m^3^ to 2187 kg/m^3^. As for workability, according to [56], it varies within one brand; however, with an increase in the percentage of replacement of part of the cement with coal dust, its decrease is observed. When the replacement value is from 4 to 6%, the consumption of the plasticizing additive increases from 1% to 1.5%; when replacing 8%, the additive consumption increases to 2%; and at 10% CD, it increases to 2.5%.

The results of studying the fresh properties of mortar mixtures are presented in Figure 6 and Figure 7.

The dependence of the density of the mortar on the CD content (%) presented in Figure 6 is described by the linear equation:

(3) $$D_{M}=2046.7-1.585\;x,\;\;\;\;\;R^{2}=0.984$$

The dependence of mortar mobility on CD content (%) presented in Figure 7 is described by the linear equation:

(4) $$M_{M}=7.44-0.218\;x,\;\;\;\;\;\;\;R^{2}=0.99$$

For the properties of fresh mortar, a similar situation is observed here as with concrete mixtures, namely, with an increase in the percentage of replacing part of the cement with coal dust, the density of the mortar mixtures also decreases slightly. The workability parameter of mortar mixtures also decreases but is within the same brand [57]. When the cement replacement rate is from 4% to 6%, the plasticizer consumption increases from 1% to 1.5%; when replacing from 8% to 10%, the additive consumption is 2%.

A decrease in the mobility of concrete and mortar mixtures when replacing part of the cement with coal dust is associated with increasing water demand. Fine particles of coal dust in concrete and mortar mixtures act as a filler. And as is known, finely dispersed mineral filler additives in cement composites can significantly increase the water–cement ratio of the mixture, which will subsequently lead to a decrease in strength characteristics. However, the use of these types of mineral additives in combination with a plasticizer will eliminate this problem and maintain the water–cement ratio and workability characteristics of the mixtures within the required limits [9]. Thus, the introduction of the plasticizing additive Sikament^®^ BV 3M into concrete mixtures and mortars with coal dust allows them to maintain their workability within the same grade.

**Figure 8:**
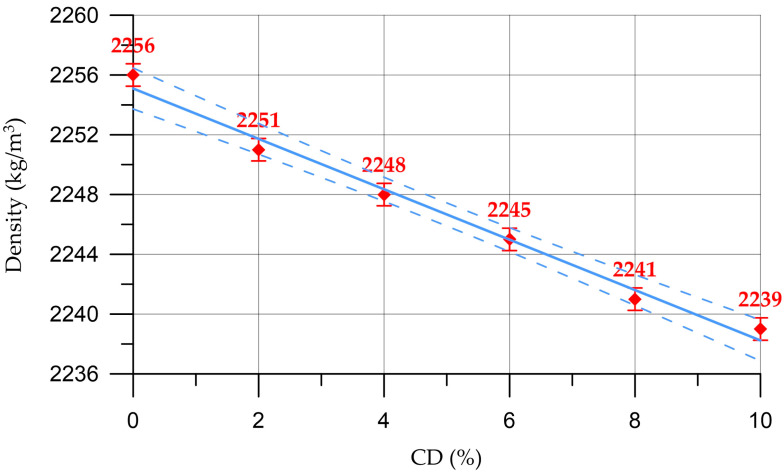
Dependence of concrete density on CD content (the dashed line shows confidence limits with a level of 0.95).

**Figure 9:**
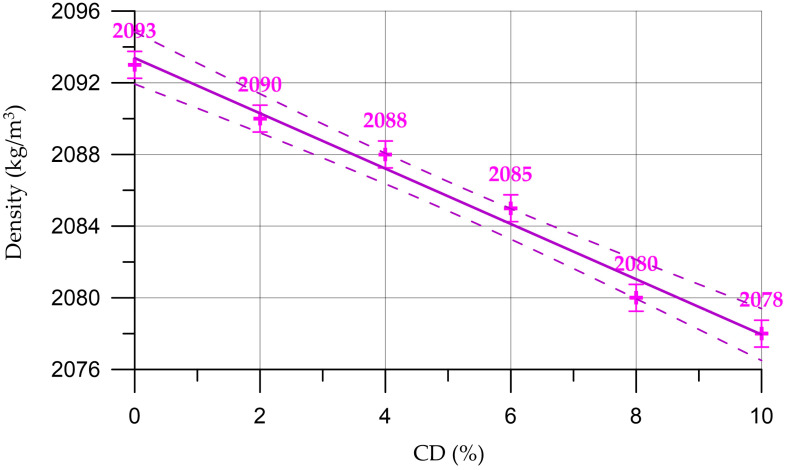
Change in mortar density depending on CD content (the dashed line shows confidence limits with a level of 0.95).

**Figure 10:**
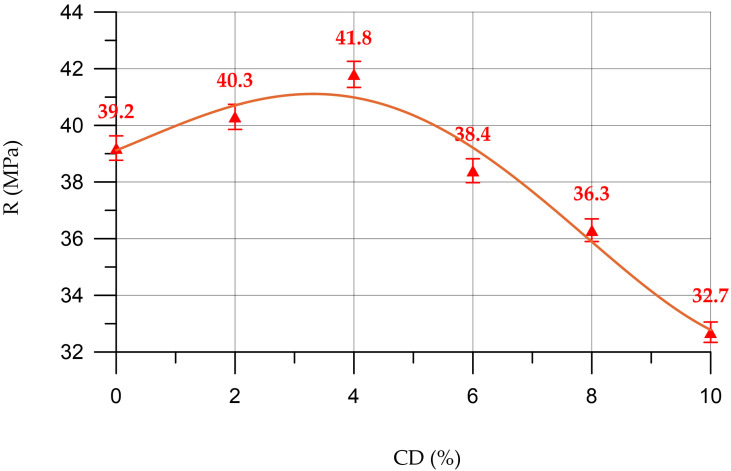
Change in compressive strength (R) of concrete depending on CD content.

**Figure 11:**
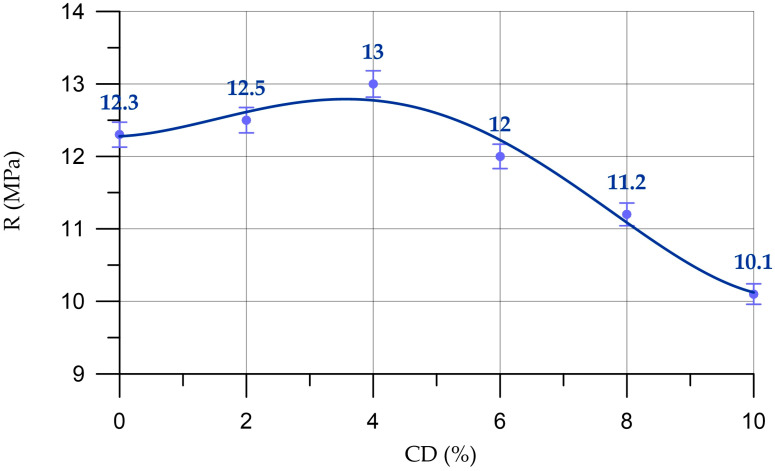
Change in compressive strength (R) of the mortar depending on the CD content.

**Figure 12:**
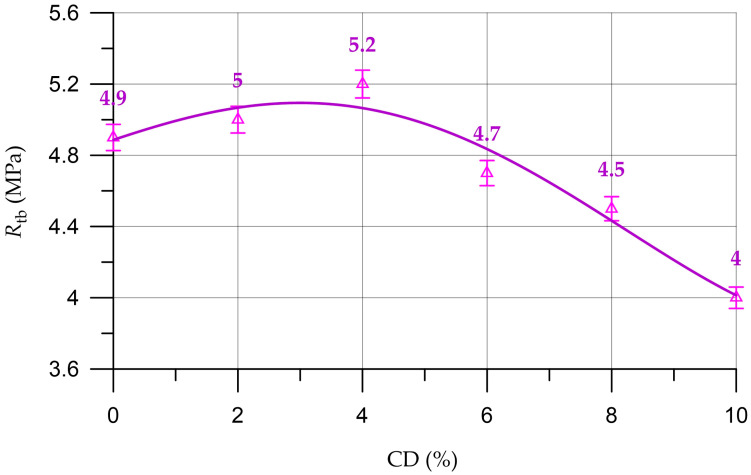
Dependence of concrete flexural strength (R_tb_) on CD content.

**Figure 13:**
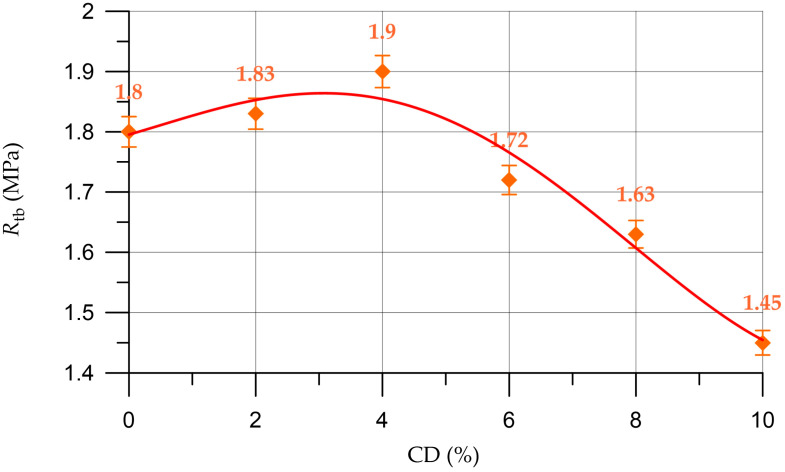
Change in the flexural strength of the mortar depending on the CD content.

The results of determining the physical and mechanical characteristics of concrete and mortars are shown in Figure 8, Figure 9, Figure 10, Figure 11, Figure 12 and Figure 13. The dependence of concrete density on the content of coal dust is presented in Figure 8.

The dependence of concrete density on CD content (%) presented in Figure 8 is described by the linear equation:

(5) $$D_{C}=\;2255-1.68\;x,\;\;\;\;R^{2}=0.987$$

Figure 9 shows the dependence of the density of the mortar on the content of coal dust.

The dependence of the density of the cement mortar on the CD content (%) presented in Figure 9 is described by the linear equation:

(6) $$D_{M}=2093-1.543\;x,\;\;\;\;\;\;\;\;R^{2}=0.984$$

From Figure 8 and Figure 9, it can be seen that the density of concrete and mortar decreases slightly with the increasing percentage of the replacement of part of the cement with coal dust. This can be explained by the fact that CD has a lower density compared to cement. However, due to small quantities, CD does not have a significant effect on the change in density of cementitious composites. Thus, the density of concrete varied from 2239 kg/m^3^ to 2256 kg/m^3^, and the density of the mortar from 2078 kg/m^3^ to 2093 kg/m^3^.

Figure 10 shows a graph of the dependence of the compressive strength of concrete on the content of coal dust in it.

To distinguish between the properties of concrete and mortar, the symbols C (for concrete) and M (for mortar) were added to the designations for equations.

The dependence of the compressive strength of concrete (*R_C_*) on CD content (%) presented in Figure 10 is described by a polynomial of the fourth degree

(7) $$R_{C}==39.11+0.84\;x+0.0727\;x^{2}-0.0545\;x^{3}+0.00325\;x^{4},\;\;\;\;\;R^{2}=0.968$$

According to the results of determining the compressive strength, presented in Figure 10, it was found that when replacing part of the cement with CD in the amounts of 2% and 4%, small increases in strength are observed up to 2.8% and 6.6%, respectively. However, with a further increase in the amount of CD, a negative effect is observed. At 6% CD, there is a slight loss in the compressive strength of 2% compared to the control composition. However, at CD quantities of 8% and 10%, significant losses in compressive strength were already recorded, which amounted to 7.4% and 16.6%, respectively.

The dependence of the compressive strength of the mortar on the content of coal dust in it is presented in Figure 11.

The dependence of the compressive strength of the mortar (*R_M_*) on CD content (%) presented in Figure 11 is described by a fourth-degree polynomial:

(8) $$R_{M}==12.27+0.041\;x+0.1192\;x^{2}-0.0314\;x^{3}+0.00169\;x^{4},\;\;\;\;\;R^{2}=0.976$$

The dependence of the compressive strength of mortar with different CD contents is similar to the change in the compressive strength of concrete. When replacing part of the cement with CD in amounts of 2% and 4%, small increases in compressive strength are observed up to 1.6% and 5.7%, respectively. At a CD quantity of 6%, a drop in compressive strength is observed by 2.4%, and at 8% and 10% CD, the compressive strength decreases by 8.9% and 17.9%, respectively.

Figure 12 shows a graph of the flexural strength of concrete.

The dependence of concrete flexural strength ($R_{tb}^{C}$) on CD content (%) presented in Figure 12 is described by a polynomial of the fourth degree:

(9) $$R_{tb}^{C}==4.88+0.1164\;x-0.0059\;x^{2}-0.00405\;x^{3}+0.0002604\;x^{4},\;\;\;\;\;R^{2}=0.95$$

The dependence of the flexural strength of concrete on the amount of CD has a similar character as the compressive strength. At a CD quantity of 2% to 4%, increases in concrete flexural strength of up to 2.0% and 6.1%, respectively, are observed, and at 6%, 8% and 10% CD, the strength decreased by 4.1%, 8.2% and 18.4%, respectively, compared to the control composition.

The dependence of the flexural strength of the mortar on the amount of coal dust is shown in Figure 13.

The dependence of the flexural strength of the mortar ($R_{tb}^{M}$) on the content of CD (%) presented in Figure 13 is described by a polynomial of the 4th degree:

(10) $$R_{tb}^{M}==1.79+0.0300\;x-0.0036\;x^{2}-0.00243\;x^{3}+0.0001432\;x^{4},\;\;\;\;\;R^{2}=0.96$$

When replacing part of the cement in the mortar with CD in an amount of 2%, the flexural strength increases by 1.7%; when replacing 4%, it increases by 5.6%; and with 6%, 8% and 10% CD, the flexural strength decreases compared to the control composition by 4.4%, 9.4% and 19.4%, respectively.

Thus, based on the results of a study of the strength characteristics of concrete and mortar with different CD contents, the following can be noted. This type of finely dispersed additive can be used as a partial replacement for cement up to 6% without a significant loss of strength characteristics. With a larger replacement of cement with coal dust, a deterioration in the strength characteristics of the composites is observed. But it is worth noting that with a quantity of coal dust from 2% to 4%, there is an increase in compressive and bending strength. This result is explained by the fact that replacing cement with 2–4% fine coal dust and adding a plasticizing additive can increase the efficiency of using CD in cement composites. Strength increases because a more dense structure of the hardened cement paste is obtained; this is achieved by increasing the volume concentration of cementing products of binder hydration and by reducing pore volume [9]. The increase in the volume concentration of cementing products of binder hydration is directly related to the chemical composition of CD, presented in Table 2. Coal dust contains 30.83% SiO_2_. The available part of silicon dioxide reacts with the hydration of the binder and promotes the formation of additional hydrosilicates [58].

**Figure 14:**
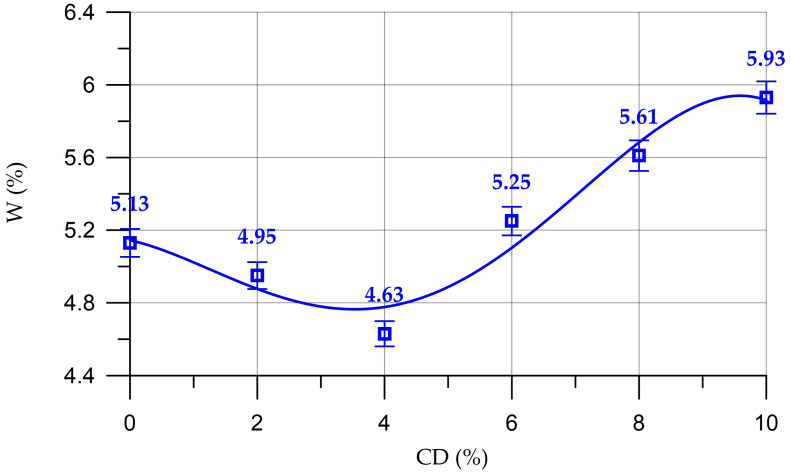
Change in water absorption of concrete depending on CD content.

**Figure 15:**
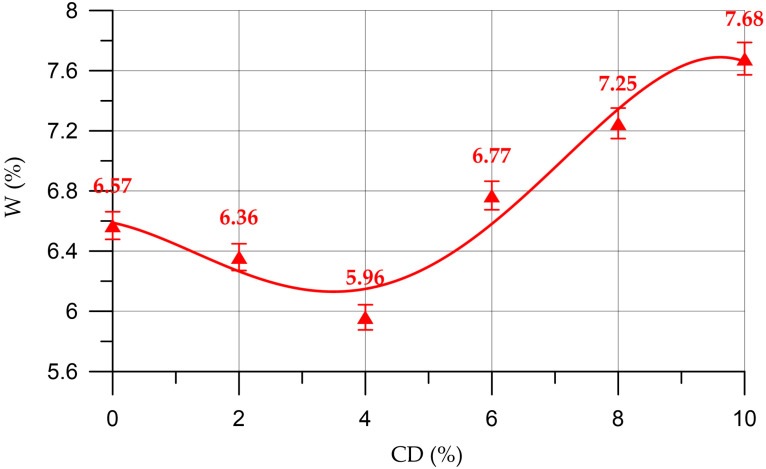
Change in water absorption of the mortar depending on the CD content.

Next, Figure 14 and Figure 15 present the results of determining the water absorption of concrete and mortar with different CD contents.

Figure 14 shows the dependence of the water absorption of concrete on the CD content.

The dependence of the water absorption of concrete on CD content (%) presented in Figure 14 is described by a fourth-degree polynomial:

(11) $$W_{C}==5.14-0.0794\;x-0.0604\;x^{2}+0.0188\;x^{3}-0.001119\;x^{4},\;\;\;\;\;R^{2}=0.95$$

According to Figure 14, the lowest water absorption values were recorded for concrete compositions with 2% and 4% CD, introduced to replace part of the cement. At these percentages of CD content, water absorption decreased by 3.5% and 9.7%, respectively. And with a CD content of 6%, 8% and 10%, the water absorption values increased by 2.3%, 9.4% and 15.6%, respectively.

Figure 15 shows a graph of the dependence of the water absorption of the mortar on the CD content in it.

The dependence of water absorption of the mortar on the content of CD (%) presented in Figure 15 is described by a polynomial of the fourth degree:

(12) $$W_{M}==6.58-0.0982\;x-0.0811\;x^{2}+0.0246\;x^{3}-0.001458\;x^{4},\;\;\;\;\;R^{2}=0.95$$

The dependence of the water absorption of the mortar on the CD content has a similar trend as the dependence of the change in water absorption of concrete. At CD quantities of 2% and 4%, the water absorption of the mortar decreased by 3.2% and 9.3%, respectively. At quantities of 6%, 8% and 10%, coal dust negatively affected the water absorption of the mortar. At 6% CD, water absorption increased by 3.0%, at 8% CD by 10.4%; and at 10% CD by 16.9%. The decrease in water absorption is associated with compaction of the structure of the hardened cement paste due to an increase in the volume concentration of cementing products of binder hydration and a decrease in pore volume [9].

Changes in the physical and mechanical characteristics of concrete and mortar with different CD contents are presented as a percentage compared to the control composition in Table 4.

**Figure 16:**
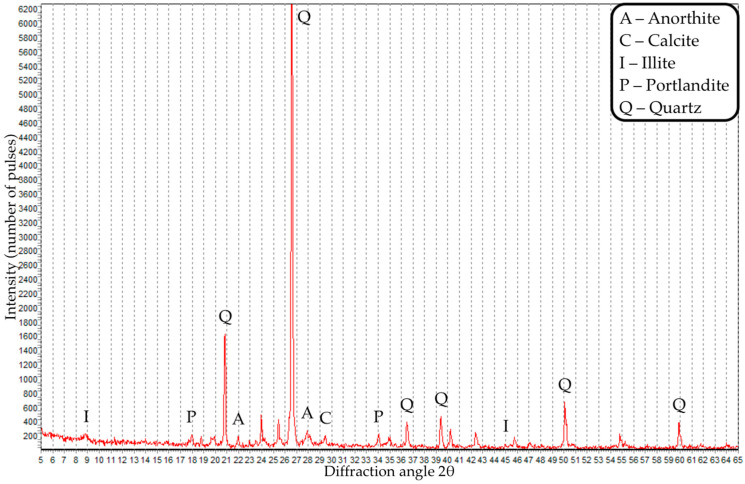
X-ray image of concrete of the control composition.

**Figure 17:**
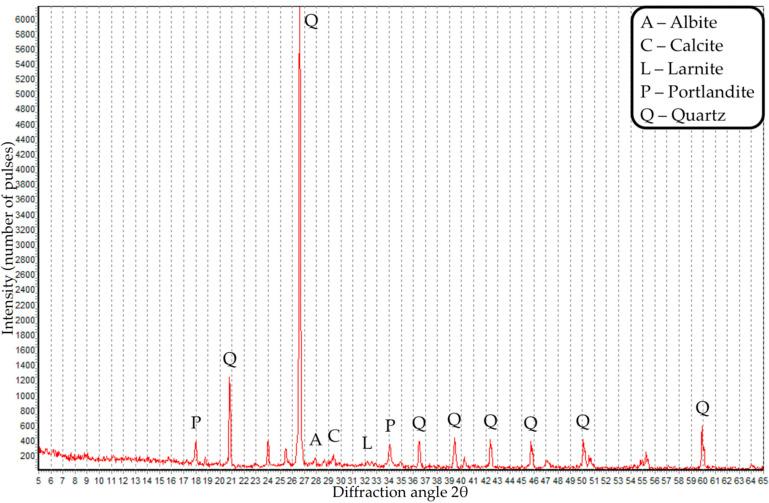
X-ray diffraction pattern of concrete with a CD content of 4%.

XRD analysis of concrete with control composition and concrete with 4% CD was also carried out. X-ray diffraction analysis of the composites is presented in Figure 16 and Figure 17.

Based on the results of XRD analysis of concrete of the control composition, the phases of portlandite, illite, anorite, calcite and quartz were identified. XRD analysis of concrete with 4% CD identified phases such as larite, albite, portlandite, calcite and quartz. Thus, the introduction of coal dust into concrete contributes to a change in its phase composition and the appearance of such phases as larite and albite.

The results obtained during this study indicate a number of achievements and need to be compared with the results of other authors obtained earlier in order to clarify the scientific novelty and scientific and practical value of this study.

So, in the course of this study, the relationships between the composition and properties of concrete and mortars based on coal dust waste were determined. The structure formation of such concrete largely depends on the following aspects. It is important to understand the role of the carbon modifier in the composition of the cement composite. If the applied carbon modifier is intended to replace part of the binder component, then, on the one hand, there is a risk of not achieving the required strength in such aspects as a decrease in the amount of binder that forms the cement coating of inert particles; an increase in fine components in the material, which additionally require wetting with water; and an increase in the wetting surface, which, in turn, leads to the risk of undercompaction and underformation of the composite structure.

Based on the results of experimental studies, it was established that with the optimal amount of replacement of part of the cement with coal dust, it is possible to obtain high-quality and environmentally efficient concrete and mortars. It should be noted that, based on the results of the literature review, no studies were found on the use of coal dust with a similar chemical composition as a partial replacement for binders in the technology of cement composites. However, there are a number of studies [13,14,15,16,17,18,19,20,21,22,23,24,25,26,27] where the use of inert mineral additives, namely, clay, metakaolin, electric arc furnace dust, stone dust and finely ground sea shell powders, as a partial replacement for cement allows in rational amounts the improvement of the physical and mechanical characteristics of the composite and at the same time an increase in its environmental friendliness. The optimal percentages for replacing part of the cement with these types of additives varied from 2% to 20%, while improvements in the strength characteristics, water absorption and porosity of experimental cement composites were observed. In this study, the optimal value for replacing part of the cement with coal dust was 4%. At the same time, the increases in compressive and bending strength were 6.6% and 6.1% for concrete and 5.7% and 5.6% for mortar compared to samples of the control composition. And the water absorption of concrete decreased by 9.7%, and for mortar it decreased by 9.3%. This is in good agreement with the works of a number of authors, in particular with the works [13,14,15,16,17,18,19] devoted to the processes of structure formation of concrete composites using coal dust and the works of the authors [20,21,22,23,24,25,26,27] devoted to the physical and mechanical characteristics of concrete modified with coal dust.

The use of coal dust of similar origin in the study [8] in small quantities—up to 2.5 wt.% of ordinary Portland cement—made it possible to improve the mechanical strength of cement composites by reducing open porosity and increased formation of hydration products compared to the reference ones. The prospect of the effective use of coal dust in appropriate, rationally selected quantities in the production of concrete was noted [8]. That study is in excellent agreement with the results presented above. In addition, the use of coal industry waste is potentially effective in the construction sector due to its mineral activity and geographical availability. Thus, separating generated waste into different fractions depending on its origin and production characteristics will be one of the innovations in the search for cleaner production and a more circular economy [10], which fits well with the points presented in this study.

Thus, in terms of replacing the binder, there are two types of risk: the risk of failure to achieve strength indicators and the risk of insufficient wetting of the inert components and hence failure to achieve uniformity of the structure [59,60,61], which affects the coefficient of structural quality [62]. As for the risks when using coal dust as a modifier in addition to the applied coarse and fine aggregates, the important role of achieving the goal of creating additional crystallization centers should be noted here. That is, coal dust in itself does not contribute to a significant improvement in the processes of structure formation, but, nevertheless, in the case of rationally selected recipe–technological parameters, it can help achieve the goal of denser packing of particles. The structural analysis, XRD analysis and confirmation of these theses by the results of laboratory physical and mechanical tests showed that the problem of determining the rational recipe and technological parameters was solved for a specific type of concrete composite using coal dust. Thus, the scientific hypothesis of this study can be considered confirmed and is in good agreement with the results previously achieved by other authors. The main value of the conducted research in a practical sense is to obtain applied experimental relationships between the composition, structure and properties of cement mortars and readymade concrete, including a large crushed stone fraction. In this regard, important parameters are the properties of cement mortars, since it is often the mortar component that is the basis for creating new types of concrete or the use of this mortar for other purposes, for example, in masonry or plastering. With all other parameters, the most important property of solutions based on coal dust is the achievement of a rational structure in order to prevent the subsequent occurrence of cracks, peeling and mechanical damage. Thus, the conducted research, in addition to its experimental value for scientific purposes, is important for the applied industry.

## 4. Conclusions

The rheological and physical–mechanical characteristics of concrete and mortar with different contents of coal dust were developed and studied. This study used normative tests and X-ray diffraction analysis. Based on the results obtained in this study, the following conclusions were drawn:

(1) It was established that coal dust, introduced to replace part of the cement, negatively affects the mobility of concrete and mortar mixtures. However, the introduction of a plasticizing additive allows one to adjust the mobility value of concrete and mortar mixtures and maintain it within the same brand.
(2) Replacing part of the cement with coal dust by up to 10% does not have a significant effect on the change in the density of concrete and mortar mixtures or in hardened composites.
(3) Compared to the control composition, concrete samples with 4% coal dust showed greater efficiency: increases in compressive and flexural strength were 6.6% and 6.1%, and water absorption decreased by 9.7%.
(4) The increases in compressive and flexural strength of the mortar with 4% coal dust content compared to control samples were 5.7% and 5.6%, and water absorption decreased by 9.3%.
(5) The most effective amount of coal dust, introduced instead of part of the cement, was 4%. It was possible to use coal dust in an amount of 6% without a significant loss of strength characteristics for both concrete and mortars.
(6) The use of coal industry waste is potentially effective in the construction sector due to its mineral activity and geographical accessibility. The prospect of effective use of coal dust in appropriate, rationally selected quantities in concrete production was confirmed.

## Figures and Tables

**Table 1:** Characteristics of raw materials.

Property					Value	
**Portland cement CEM I 52.5N**						
Specific surface area (m^2^/kg)					339	
Soundness (mm)					0.5	
Fineness, passage through sieve No. 008 (%)					97.5	
Setting times (min): - Start - End					170 240	
Compressive strength (MPa): - 2 days - 28 days					25.7 58.5	
**Crushed sandstone**						
Particle size (mm)					5–20	
Bulk density (kg/m^3^)					1429	
Apparent density (kg/m^3^)					2553	
Resistance to fragmentation (wt %)					11.8	
Content of lamellar and acicular grains (wt %)					9.7	
**Quartz sand**						
Sieve diameter (mm)					Content (% by weight) of grains with a particle size of less than 0.16 mm	Fineness modulus
Partial residues on sieves (%)						
Total residues on sieves (%)						
2.5	1.25	0.63	0.315	0.16		
3.2	10.1	14.3	16.6	54.7	1.0	1.87
3.2	13.3	27.6	44.2	99.0		
Bulk density (kg/m^3^)					1255	
The content of dust and clay particles (%)					0.09	
Content of clay in lumps (%)					0.12	
Organic and contaminant content (%)					No	
**Coal dust (CD)**						
Bulk density (kg/m^3^)					345	
Loss on ignition (%)					37.34	
Silicon oxide SiO_2_ (%)					30.83	
Aluminum oxide Al_2_O_3_ (%)					15.74	
Iron oxide Fe_2_O_3_ total (%)					6.22	
Calcium oxide CaO (%)					2.92	
Magnesium oxide MgO (%)					3.43	
Titanium oxide TiO_2_ (%)					0.64	
Phosphorus oxide P_2_O_5_ (%)					0.07	
Total sulfur oxide SO_3_ (%)					2.81	

**Table 2:** Concrete mix designs.

Mixture Type	Concrete Mixture Proportion per 1 m^3^					
	C (kg/m^3^)	W (L/m^3^)	CS (kg/m^3^)	S (kg/m^3^)	CD (kg/m^3^)	P (% by Weight of Cement)
0CD/C	353	199	936	711	0	0
2CD/C	345.9	199	936	711	7.1	1.0
4CD/C	338.9	199	936	711	14.1	1.5
6CD/C	331.8	199	936	711	21.2	1.5
8CD/C	324.8	199	936	711	28.2	2.0
10CD/C	317.7	199	936	711	35.3	2.5

**Table 3:** Mortar mix designs.

Mixture Type	Mortar Mixture Proportion per 1 m^3^				
	C (kg/m^3^)	W (L/m^3^)	S (kg/m^3^)	CD (kg/m^3^)	P (% by Weight of Cement)
0CD/M	346	214	1481	0	0
2CD/M	339.1	214	1481	6.9	1.0
4CD/M	332.2	214	1481	13.8	1.5
6CD/M	325.2	214	1481	20.8	1.5
8CD/M	318.3	214	1481	27.7	2.0
10CD/M	311.4	214	1481	34.6	2.0

Note: C—cement, W—water, CS—crushed stone, S—sand, CD—coal dust, P—plasticizer.

**Table 4:** Changes in the physical and mechanical characteristics of concrete and mortar with different CD contents.

Characteristics	∆ (%) with CD Content Introduced Instead of Part of the Cement (%)					
	0	2	4	6	8	10
**Concrete**						
Density (kg/m^3^)	0	−0.2	−0.4	−0.5	−0.7	−0.8
R (MPa)	0	2.8	6.6	−2.0	−7.4	−16.6
R_tb_ (MPa)	0	2.0	6.1	−4.1	−8.2	−18.4
W (%)	0	−3.5	−9.7	2.3	9.4	15.6
**Mortar**						
Density (kg/m^3^)	0	−0.1	−0.2	−0.4	−0.6	−0.7
R (MPa)	0	1.6	5.7	−2.4	−8.9	−17.9
R_tb_ (MPa)	0	1.7	5.6	−4.4	−9.4	−19.4
W (%)	0	−3.2	−9.3	3.0	10.4	16.9

## Data Availability

The study did not report any data.
